# Detraining Effect on Pulmonary and Cardiovascular Autonomic Function and Functional Outcomes in Patients With Parkinson's Disease After Respiratory Muscle Training: An 18-Month Follow-Up Study

**DOI:** 10.3389/fneur.2021.735847

**Published:** 2021-10-21

**Authors:** Chih-Cheng Huang, Yun-Ru Lai, Fu-An Wu, Nai-Ying Kuo, Ben-Chung Cheng, Nai-Wen Tsai, Chia-Te Kung, Yi-Fang Chiang, Cheng-Hsien Lu

**Affiliations:** ^1^Department of Neurology, Kaohsiung Chang Gung Memorial Hospital, Chang Gung University College of Medicine, Kaohsiung, Taiwan; ^2^Department of Respiratory Therapy, Kaohsiung Chang Gung Memorial Hospital, Chang Gung University College of Medicine, Kaohsiung, Taiwan; ^3^Department of Internal Medicine, Kaohsiung Chang Gung Memorial Hospital, Chang Gung University College of Medicine, Kaohsiung, Taiwan; ^4^Department of Emergency Medicine, Kaohsiung Chang Gung Memorial Hospital, Chang Gung University College of Medicine, Kaohsiung, Taiwan; ^5^Center for Shockwave Medicine and Tissue Engineering, Kaohsiung Chang Gung Memorial Hospital, Chang Gung University College of Medicine, Kaohsiung, Taiwan; ^6^Department of Biological Science, National Sun Yat-sen University, Kaohsiung, Taiwan; ^7^Department of Neurology, Xiamen Chang Gung Memorial Hospital, Xiamen, China

**Keywords:** respiratory muscle training, detraining effect, pulmonary function, cardiovascular autonomic function, Parkinson's disease

## Abstract

**Background:** The effect of 3-month respiratory muscle training (RMT) on pulmonary and autonomic function and functional outcomes has been demonstrated in patients with Parkinson's disease (PD); however, there is a paucity of information on the durability of the training effect. In this study, we monitored the pulmonary and cardiovascular autonomic function and clinical severity scales until 18 months after the cessation of RMT to elucidate the detraining effect after RMT.

**Methods:** All patients with PD receiving RMT were assessed with clinical severity scales as well as pulmonary and autonomic function tests at four different stages (baseline on enrollment, immediately after 3 months of RMT, and 6 and 18 months after cessation of RMT). A control group of PD patients who did not receive RMT was also recruited for comparison. Pulmonary function parameters, including forced vital capacity (FVC), forced expiratory volume in one second (FEV1), maximum inspiratory pressure (MIP), and maximum expiratory pressure (MEP), were assessed. Cardiovascular autonomic function was assessed using measures including heart rate response to deep breathing (HRDB), Valsalva ratio, and baroreflex sensitivity. Clinical severity scores were also measured using the Hoehn and Yahr staging and the Unified Parkinson's Disease Rating Scale (UPDRS).

**Results:** The results showed significant improvements in MIP, MEP, HRDB, and UPDRS immediately after RMT. Despite some decay, the improvements in pulmonary function (MIP and MEP) and functional outcomes (UPDRS) remained significant until 6 months of detraining (9 months after enrollment). However, the improvement in cardiovascular autonomic function (HRDB) was reversed after 6 months of detraining.

**Conclusions:** Based on these findings, we recommend that RMT may be repeated after at least 6 months after previous session (9 months after enrollment) for patients with PD to maintain optimal therapeutic effects.

## Introduction

Patients with Parkinson's disease (PD) commonly present with restrictive pulmonary dysfunction. Decreased respiratory muscle strength and endurance with increased dyspnea are observed in ~50–60% of patients with PD (1, 2), although their pulmonary disorders usually remain unnoticed until advanced stages of the disease (1–3). Previous studies have shown the effect of respiratory muscle training (RMT) on pulmonary function, swallowing function, and quality of life (4–6).

The autonomic nervous system is known to play a pivotal role in the regulation of lung ventilation, gas exchange, and smooth muscle function of the airway (7). Autonomic impairment is also an important non-motor feature of PD (8, 9). Furthermore, the observation that levodopa has limited effects on the improvement of pulmonary function in patients with PD may suggest that respiration is associated with motor function, which is mainly mediated by the dopaminergic system, and other networks (10–15). Breathing involves a complex reciprocal interaction of the respiratory neuron network (RNN) and the central autonomic network (CAN) (16), and these two networks interact harmoniously to maintain adequate respiratory function. Our previous study showed that there was a simultaneous improvement in pulmonary and cardiovascular autonomic function in patients with PD after RMT (17).

Although the short-term effect of RMT on pulmonary and autonomic functions has been demonstrated, there is a paucity of information on the durability of the training effect. In the current study, we monitored pulmonary and autonomic functions up to 18 months after the cessation of RMT in patients with PD and attempted to elucidate the detraining effect. These approaches to improve the long-term outcome and quality of life of patients with PD in clinical practice are promising.

## Patients and Methods

### Study Design and Participants

This single-center prospective case-control study was conducted at a tertiary medical center and the main referral hospital in southern Taiwan. We evaluated patients with a definitive diagnosis of idiopathic PD based on the clinical diagnostic criteria as well as the magnetic resonance imaging findings (18, 19) in our previous study (17). The exclusion criteria were (1) newly diagnosed with PD or were on follow-up for <6 months, (2) presence of focal neurological signs not related to the diagnostic criteria of PD, (3) having diabetes, (4) active smoker or had quit <5 years ago, (5) obstructive type pulmonary diseases, which was defined as FE *V1*/FVC <0.7 (20), (6) suffered from moderate-to-severe heart failure (NYHA classes III and IV), and (7) having any type of arrhythmia that prevented BRS measurement, or pacemaker implantation due to any cause. Their pulmonary and autonomic functions were monitored, and we compared them with an age-, sex-, and body mass index (BMI)-matched control group for 18 months after RMT. Some of the patients in the RMT and control groups were lost to follow-up or quit before the completion of the study. Thus, only 32 patients who received RMT, as well as 20 cases who did not, presented for complete follow-up after 18 months after RMT. All participants received verbal and written information about the purpose and methodology of our research, which was approved by the Institutional Review Committees on Human Research of the hospital (201702037B0D001).

### Study Protocol

**Figure 1:**
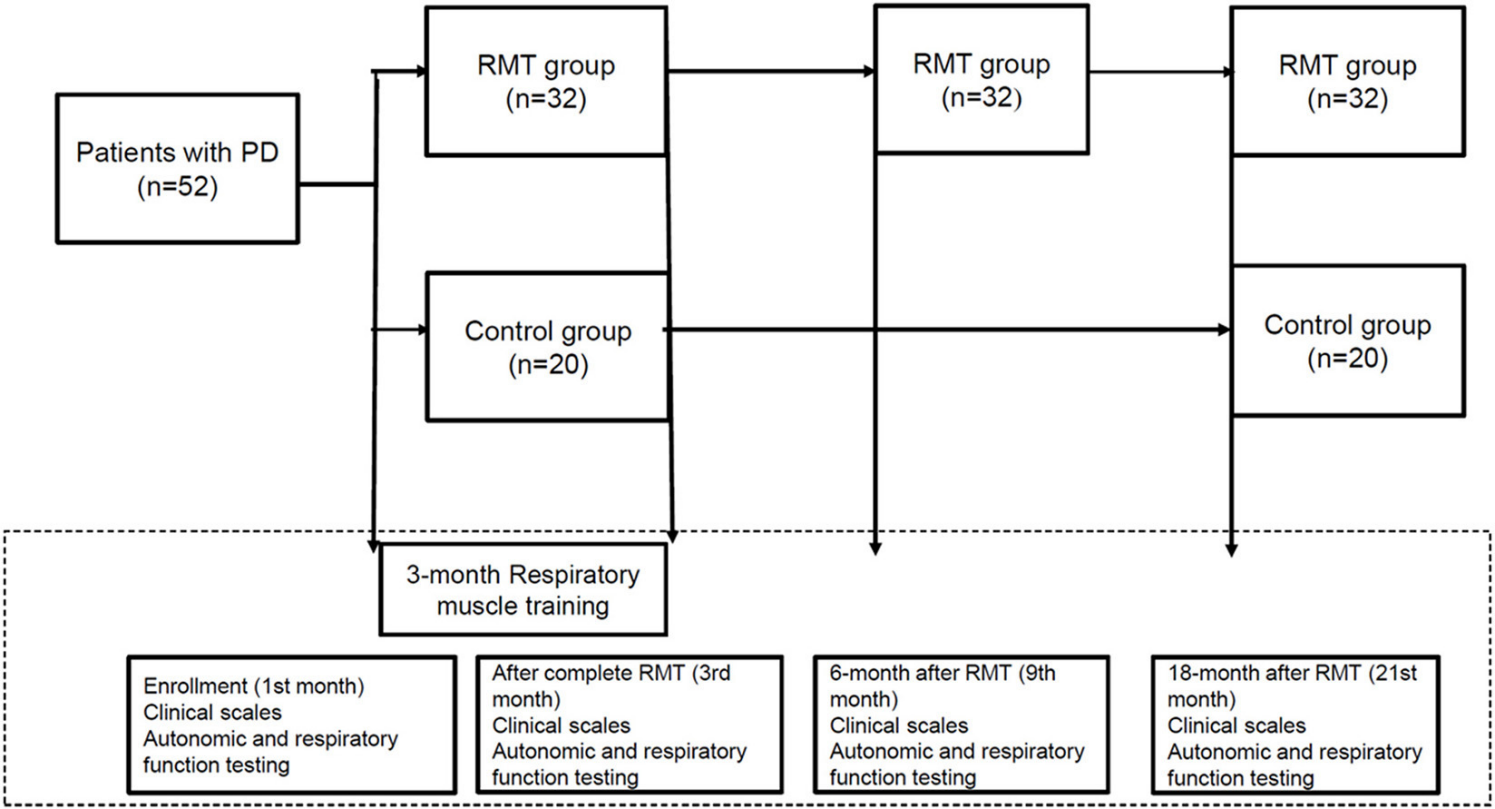
Flow chart of the study protocol.

Figure 1 is the flow chart of the study protocol. The enrolled participants in the RMT group were assessed using clinical scales and pulmonary and autonomic testing four times during the study period: at baseline upon enrollment, immediately after 3 months of RMT, and 6 and 18 months after the cessation of RMT. The control group was only assessed thrice during the study period (at baseline on enrollment, immediately after 3 months of RMT, and 18 months after the cessation of RMT). These assessments, including respiratory and autonomic function testing and clinical scales, were performed during the “off” period of medication, which was defined as 8–12 h after the latest dose of anti-parkinsonism treatment.

### Respiratory Muscle Training

The RMT protocol was consistent with those of previous studies (17, 21, 22). RMT was performed by an experienced respiratory therapist using a Dofin Breathing Trainer for 30 min twice daily for at least 5 days a week for 3 months.

### Clinical Assessment

The following clinical features were recorded upon enrollment: age, sex, height, weight, body mass index, disease duration, levodopa equivalent dose (LED) (23), and clinical severity scores using the Unified Parkinson's Disease Rating Scale (UPDRS) and the Hoehn and Yahr (H-Y) staging (21, 22). *One* neurologist (C.C.H) evaluates the UPDRS scores and H-Y staging blind to the patients.

### Pulmonary and Autonomic Function Testing

The pulmonary function of every patient with PD was evaluated using spirometry without exposure to a bronchodilator according to the guidelines of the American Thoracic Society (17, 24). These recorded parameters included forced vital capacity (FVC), forced expiratory volume in one second (FEV1), MIP, and MEP.

A standardized evaluation of cardiovascular autonomic function, as described by Low (25), was done and the tests included deep breathing and the Valsalva maneuver (VM). Baroreflex sensitivity (BRS) was determined based on the changes in heart rate and BP during the early phase II of VM by applying least-squares regression analysis.

### Statistical Analysis

The categorical variables were compared using the chi-squared or Fisher's exact tests. Continuous data are expressed as the mean ± standard deviation (SD) or median [interquartile range (IQR)]. Those continuous data that were not normally distributed were logarithmically transformed to improve normality and compared using Student's *t*-tests. The parameters of the respiratory and cardiovascular autonomic functions and the functional scores at baseline and 3, 6, and 18 months post-RMT were compared using a paired *t*-test. Repeated-measures analysis of variance was used to compare the parameters of the respiratory and cardiovascular autonomic functions and functional scores at different stages of the study (baseline, 3 months, and 18 months post-RMT). Analysis of covariance (ANCOVA) was used to compare the biomarkers of the RMT subgroups and controls, with sex and age as potential confounding variables. The Levene test of equality of error variance was used to ensure equal variance in both groups. All statistical analyses were conducted using the Statistical software (IBM SPSS Statistics v23, IBM, Redmond, WA, USA).

## Results

### Baseline Characteristics of the Patients

Table 1 shows the characteristics and baseline parameters of the pulmonary and cardiovascular autonomic functions of the control (*n* = 20) and RMT (*n* = 32) groups. In the control group, 8 patients had restrictive pattern of pulmonary function, and 12 patients had normal pattern. In the RMT group, 12 patients had restrictive pattern, and the other 20 patients had normal pattern. The distribution in terms of the pulmonary function was similar between groups. There was no significant difference between the two groups related to age, sex, duration of disease, or LED. The difference between the H-Y stages of the groups reached borderline significance (slightly higher in the control group). For the UPDRS, there was no significant difference between the groups related to subscores I, II, III, or total score.

**Table 1:** Comparison of demographic and laboratory data between groups of disease control subjects and patients with RMT.

	**Controls**	**RMT group**	***P*-value**
	**(*n* = 20)**	**(*n* = 32)**	
Age	66.3 ± 9.8	63.5 ± 9.9	0.245
Gender (female/male)	10/10	19/13	0.617
Disease duration (year)	6.0 [2.0, 9.0]	4.5 [2.0, 8.0]	0.490
Levodopa equivalent dose (mg)	777 ± 423	716 ± 490	0.581
H-Y stage	2.5 [2.0, 3.0]	2.0 [1.5, 2.5]	0.047^*^
UPDRS subscore I	2 [1, 3]	2 [1, 3]	0.472
UPDRS subscore II	10 [8, 13]	10 [4, 12]	0.551
UPDRS subscore III	26 [17.5, 37.5]	21 [10, 29]	0.139
UPDRS total	40 [29, 52]	34 [18, 43]	0.151
**Medication^Φ^**
Dopamine agonist	8/20 (40%)	14/32 (43.8%)	0.70
Selegiline/Rasagiline	5/20 (25%)	10/32 (31.3%)	0.235
Entacapone	7/20 (35%)	9/32 (28.1%)	0.665
Anti-psychotics	2/20 (10%)	3/32 (9.3%)	1.0
BZD	7/20 (35%)	11/32 (34.4%)	1.0
Anti-hypertension	7/20 (35%)	12/32 (37.5%)	1.0

* *P < 0.05, independent-t test; RMT, respiratory muscle training; H-Y, Hoehn and Yahr; UPDRS, Unified Parkinson's Disease Rating Scale; BZD, benzodiazepam;*

Φ *All the patients took more than one kinds of anti-Parkinsonian medications*.

### Serial Changes in Pulmonary Function Parameters and Autonomic Parameters Between the Control and Respiratory Muscle Training Groups

**Figure 2:**
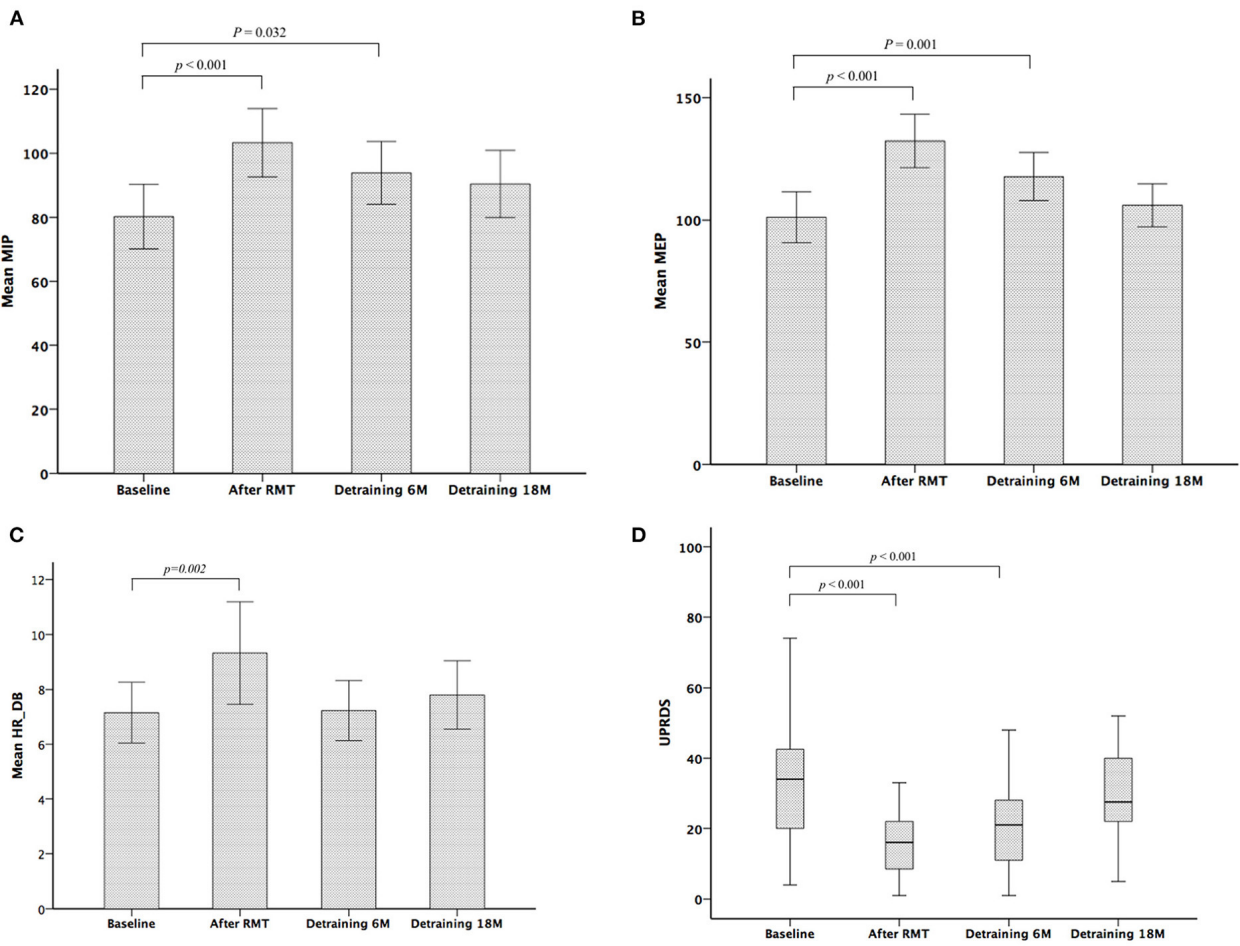
Serial changes in maximum inspiratory pressure (MIP) **(A)**, maximum expiratory pressure (MEP) **(B)**, heart rate response to deep breathing (HRDB) **(C)**, and Unified Parkinson's Disease Rating Scale (UPDRS) **(D)** during the study period.

Table 2 shows the parameters of pulmonary and autonomic function for the two groups of patients at three time points: baseline, 3rd month (immediately after treatment for the RMT group), and 21st month (18 months after cessation of treatment for the RMT group). In the RMT group, there were significant improvements in pulmonary function, as indicated by the MIP and MEP, and cardiovascular autonomic function, as indicated by the HRDB in the 3rd month (just after RMT treatment). As Figure 2 shows, the improvement in pulmonary function decayed, but it remained significant 6 months after the cessation of RMT. The improvement in HRDB became non-significant 6 months after the cessation of RMT. At the 21st month (18 months after cessation of RMT), all the parameters of the pulmonary and cardiovascular autonomic functions returned to values similar to baseline. No improvement was observed in the control group. In contrast, there was a significant deterioration in FEV1 and FEV1/FVC in the control group (*p* = 0.011 and 0.020, respectively, *paired t-test*) the 3rd month after RMT. Repeated-measures ANOVA was performed to compare the changes in the three different time periods (baseline, 3 months, and 18 months post-RMT) in these two groups, and the results of the statistical analyses showed only MEP (*p* = 0.045) researched statistical significance, but the other parameters did not. The statistical results in the other parameters were as follows: HRDB (*p* = 0.927), VR (*p* = 0.406), BRS_seq (*p* = 0.106), FVC (% pred) (*p* = 0.927), FEV1 (% pred) (*p* = 0.406), and MIP (*p* = 0.74).

**Table 2:** Comparison of serial changes of parameters in pulmonary function and autonomic function between two groups.

	**Baseline**		**3 months after RMT**		**18 months after RMT**	
	**Controls**	**RMT group**	**Controls**	**RMT group**	**Controls**	**RMT group**
	**(*n* = 20)**	**(*n* = 32)**	**(*n* = 20)**	**(*n* = 32)**	**(*n* = 20)**	**(*n* = 32)**
**Parameters of pulmonary function**
FVC (% pred)	85.8 ± 19.5	85.7 ± 16.7	82.9 ± 19.3	81.5 ± 13.5	84.1 ± 17.6	81.5 ± 12.0
FEV1 (% pred)	88.2 ± 21.0	86.3 ± 13.8	80.7 ± 20.9^*^	84.3 ± 15.8	81.3 ± 19.0	84.8 ± 12.7
FEV1/FVC (%)	81.5 ± 8.3	80.9 ± 9.2	77.2 ± 11.0^*^	81.7 ± 8.0	76.5 ± 9.1	82.1 ± 6.2
MIP	92.8 ± 37.3	80.3 ± 31.5	97.3 ± 35.6	103.3 ± 33.2^*^	73.3 ± 35.8^*^	90.5 ± 30.2
MEP	91.0 ± 32.3	101.1 ± 32.6	95.3 ± 37.9	132.3 ± 34.1^*^	85.3 ± 34.2	106.1 ± 25.3
**Parameters of autonomic function**
HRDB	6.9 ± 3.4	7.2 ± 3.4	7.2 ± 4.0	9.3 ± 5.9^*^	7.3 ± 4.1	7.8 ± 3.5
Valsalva ratio	1.29 ± 0.19	1.35 ± 0.15	1.28 ± 0.15	1.39 ± 0.24	1.22 ± 0.10	1.33 ± 0.15
BRS	1.7 ± 0.9	1.8 ± 0.9	1.8 ± 0.8	2.1 ± 1.2	1.4 ± 1.1	1.9 ± 0.9

* *P < 0.05, by comparison with the data before treatment, paired-t test*.

*RMT, respiratory muscle training; FVC, forced vital capacity; FEV1, forced expiratory volume in one second; MIP, maximum inspiratory pressure; MEP, maximum expiratory pressure; HRDB, heart rate response to deep breathing; VR, Valsalva ratio; BRS, baroreflex sensitivity*.

### Serial Changes in Clinical Severity Scales in the Control and RMT Groups During the Study Period

Table 3 shows the scores of functional outcomes for the two groups of patients at three time points: baseline, 3rd month (immediately after treatment for the RMT group), and 21st month (18 months after cessation of treatment for the RMT group). In the RMT group, a significantly improved functional status was observed in the UPDRS (including subscores I, II, III, and total score) after RMT. The improved functional status remained significant 6 months after the cessation of RMT, but it became non-significant and returned to baseline 18 months after the cessation of RMT (Figure 2). In the control group, the functional outcome scores did not change significantly throughout the study period. However, repeated-measures ANOVA was performed to compare the changes in the three different time periods (baseline, 3 months, and 18 months post-RMT) between these two groups and showed no significant statistical difference. The statistical analyses were as follows: UPDRS subscore I (*p* = 0.091), II (*p* = 0.236), III (*p* = 0.239), and total scores (*p* = 0.186). There was significant increment of LED in both control and RMT groups at the 18 *months* after cessation of RMT compared with that at baseline, implying that there was disease progression in these patients during the follow-up period. The change in LED was larger in the control group than in the RMT group, although the difference was not significant.

**Table 3:** Comparison of serial changes of functional scores between two groups.

	**Baseline**		**3 months after RMT**		**18 months after RMT**	
	**Controls**	**RMT group**	**Controls**	**RMT group**	**Controls**	**RMT group**
	**(*n* = 20)**	**(*n* = 32)**	**(*n* = 20)**	**(*n* = 32)**	**(*n* = 20)**	**(*n* = 32)**
H-Y stage	2.5 [2.0, 3.0]	2.0 [1.5, 2.5]	2.5 [1.5, 3.0]	2.0 [1.5, 2.0]	2.5 [2.0, 3.0]	2.0 [2.0, 2.6]
UPDRS subscore I	2 [1, 3]	2 [1, 3]	2.0 [1.0, 3.0]	1.0 [1.0, 2.0]^*^	2.0 [1.0, 5.0]	1.0 [1.0, 3.0]
UPDRS subscore II	10 [8, 13]	10 [4, 12]	9.0 [4.0, 13.3]	6.0 [2.0, 9.0]^*^	10.0 [6.0, 13.0]	9.0 [6.8, 12.3]
UPDRS subscore III	26 [17.5, 37.5]	21 [10, 29]	23.0 [10.8, 27.3]	8.0 [5.0, 12.0]^*^	23.0 [16.0, 31.0]	17.5 [12.5, 26.3]
UPDRS total	40 [29, 52]	34 [18, 43]	34.5 [16.8, 44.0]	16.0 [8.0, 22.0]^*^	37.0 [24.0, 51.0]	27.5 [21.5, 40.3]
LED (mg/day)	777 ± 423	716 ± 490	777 ± 423	716 ± 490	1,190 ± 716	1,015 ± 636

* *P < 0.05, by comparison with the data before treatment, paired-t test*.

*RMT, respiratory muscle training; H-Y, Hoehn and Yahr; UPDRS, Unified Parkinson's Disease Rating Scale; LED, levodopa equivalent dose*.

## Discussion

To date, there is paucity of information on the detraining effect after RMT, although the treatment effects of RMT in patients with PD have been demonstrated in our previous study, as well as several previous reports (4–6, 17). The detraining effect after RMT on pulmonary function has been reported in a study conducted in patients with late-onset Pompe disease (LOPD) (26). Concerning the detraining effect on cardiovascular autonomic function after RMT, only a recent study was conducted in healthy older women (27). To the best of our knowledge, the current study is the first to address the detraining effect after RMT on pulmonary and cardiovascular autonomic functions in patients with PD for a longer period.

The improvements in pulmonary and cardiovascular autonomic functions decayed after the cessation of RMT. According to the current data, the former decay is slower than the latter. This seems reasonable because the effect of RMT on pulmonary function is direct, whereas the effect on cardiovascular autonomic function is indirect and less prominent. The improvement of pulmonary function, as indicated by MIP and MEP, remained significant until 6 months after the cessation of RMT (9 months after enrollment). The study by Jones et al. was performed in patients with LOPD and showed that the RMT effect was durable after 3 months of detraining (26). Their study did not have data for longer than 3 months of detraining. Regarding cardiovascular autonomic function, our data showed that the treatment effect lasted for less than 6 months. The effect lasted for much less than 6 months according to the study by Rodrigues et al., which revealed that the improvements in vagal function and heart rate variability induced by RMT were reversed by 4 weeks of detraining, although their study involved older healthy women (27).

Regarding the functional score, the H-Y stage was consistent throughout the study in both the RMT and control groups. The H-Y stage may not be sensitive enough to reflect the change in functional outcomes in these patients. There was significant improvement in UPDRS (subscores II, III, and total score) after RMT. All the patients enrolled in this study were on follow-up for more than 6 *months*, and their daily dose of anti-Parkinsonian agents was at a steady dosage. The antiparkinsonian medications were not changed during the 3 *months* of RMT. Thus, the improvement in UPDRS is likely due to RMT rather than medication change. The improvement in UPDRS (UPDRS subscore II, UPDRS subscore III, and UPDRS total score) between baseline and 6-*month* follow-up (3 *months* after RMT) did not show statistical difference among the *three* motor phenotypes (*p* = 0.127, *p* = 0.279, and *p* = 208, *respectively*. Repeated measure A*NOVA*). The improvement in UPDRS remained significant until 6 months after the cessation of RMT (9 months after enrollment) and was reversed by 18 months of detraining. The detraining curve of UPDRS was similar to that of pulmonary function, rather than cardiovascular autonomic function. This finding may be consistent with our findings because daily activity and motor function depend more on pulmonary function than on cardiovascular autonomic function. Autonomic impairment usually remains asymptomatic until advanced stages.

The main limitation of this study is that protocol adherence was not as good in the control group. Although our previous study allocated 37 patients to the control group (17), it led to a reduction in the number of cases in the control group during the follow-up. Only 20 patients in the control group completed the entire study. The most common reason for the inability of patients to continue with the protocol was worse daily function. The deterioration of daily function in those patients during the follow-up was so marked that they were unwilling to report to the hospital for the entire set of evaluations. Therefore, if those patients had been included for the assessments during the 9th and 21st months, the data of the control group would have become even worse. In contrast, 32 of the 38 patients in the RMT group completed the study protocol. Considering that the baseline conditions were similar in these two groups of patients, the functional deterioration was slower in the patients with RMT. In addition, some may argue that there were older age, more disease severity, and longer duration in the control group. Although the age, and disease severity and duration were not perfectly matched between control and study groups, we had done our best to do so, and the difference was not statistically significant.

## Conclusion

This study demonstrated the detraining effect of RMT in patients with PD. The improvement in pulmonary function and functional outcomes may persist for at least 6 months after detraining. However, the effect on cardiovascular autonomic function was less than 6 months. Based on these findings, we recommend that RMT may be repeated at least 6 months after previous session for patients with PD to maintain optimal therapeutic effects.

## Data Availability Statement

The raw data supporting the conclusions of this article will be made available by the authors, without undue reservation.

## Ethics Statement

The study was conducted according to the guidelines of the Declaration of Helsinki, and approved by the Institutional Review Board of Chang Gung Medical Foundation (201702037B0D001). The patients/participants provided their written informed consent to participate in this study.

## Author Contributions

C-CH and C-HL conceptualized the study, developed the methodology, wrote, reviewed, and edited the manuscript. C-CH, Y-RL, F-AW, N-YK, B-CC, N-WT, and C-HL performed the formal analysis. Y-RL, F-AW, N-YK, B-CC, and N-WT conducted the investigation. C-HL provided the resources, supervised the study, was in charge of the project administration, and acquired the funding. C-CH wrote and prepared the original draft. All authors have read and agreed to the published version of the manuscript.

## Funding

This study was supported by Grants from the Ministry of Science and Technology (MOST 106-2314-B-182A-072 and 107-2314-B-182A-046-MY2).

## Conflict of Interest

The authors declare that the research was conducted in the absence of any commercial or financial relationships that could be construed as a potential conflict of interest.

## Publisher's Note

All claims expressed in this article are solely those of the authors and do not necessarily represent those of their affiliated organizations, or those of the publisher, the editors and the reviewers. Any product that may be evaluated in this article, or claim that may be made by its manufacturer, is not guaranteed or endorsed by the publisher.
